# Responsibilities

**Published:** 1869-04

**Authors:** 


### RESPONSIBILITIES.

An eminent clergyman in a religious newspaper refers, in strong terms, "to the responsibility of giving our names to recommend men, or measures, or books;" but if these are great, being mere matters of opinion as to character, or involving a few dollars and cents, it seems to be a greater responsibility which editors assume when they insert among their reading matter, for pay, as an advertisement, as if it were selected for its general value, the recommendation of some patent medicine, the constituents of which they do not certainly know; and if they did, have no accurate scientific knowledge of the effects on the human system, present and remote; and yet, in the very excellent paper referred to, there has often appeared the personal recommendation of the editor relative to different patent medicines, two of which follow:

"Public Speakers and Singers will find *Troches* beneficial in clearing the voice before speaking or singing, and relieving the throat after any unusual exertion of the vocal organs, having a peculiar adaptation to affections which disturb the organs of speech. For Coughs and Colds ——'s *Troches* are effectual."

"FOUND AT LAST! A remedy that not only relieves, but cures that enemy of mankind, Consumption, as well as the numerous satellites which revolve around it in the shape of Coughs, Colds, Bronchitis, Sore Throat, Influenza, etc. The remedy we allude to is prepared in Boston."

In the first advertisement, or rather "announcement," there is no indication that it is anything else than an assurance of the editor that carries the impression to the casual reader, that the "Troches" cure coughs and colds. Is it so? We should think that any man, even of moderate intelligence, would know that any medicine which would cure coughs and colds, would become world-renowned in a year, without the aid of continuous advertisements; and would make the person who owned the secret the richest man on the globe in a few years.

The next announcement "cures consumption!" and goes on to say that "the remedy we allude to," etc. When the word "we" is used in a newspaper, it implies that the editor is the individual who speaks. We do not wish to put ourselves in wrong position by using epithets, but simply ask the question, ought the editor of a religious newspaper, or the publisher, tell a whopper as big as nineteen barns for two or three dollars?

